# Vinflunine: a new active drug for second-line treatment of advanced breast cancer. Results of a phase II and pharmacokinetic study in patients progressing after first-line anthracycline/taxane-based chemotherapy

**DOI:** 10.1038/sj.bjc.6603347

**Published:** 2006-10-10

**Authors:** M Campone, H Cortes-Funes, D Vorobiof, M Martin, C F Slabber, E Ciruelos, E Bourbouloux, C Mendiola, F M Delgado, C Colin, V Aslanis, P Fumoleau

**Affiliations:** 1 Centre René Gauducheau, 44805 Saint Herblain, France; 2 Hospital 12 de Octubre, 28041 Madrid, Spain; 3 Sandton Oncology Centre, Sandton, South Africa; 4 Hospital Universitario San Carlos, Madrid, Spain; 5 Pretoria Academic Hospital, Pretoria, South Africa; 6 Institut de Recherche Pierre Fabre, 92654 Boulogne-Billancourt, France

**Keywords:** advanced breast cancer, vinflunine

## Abstract

To evaluate the single agent activity, pharmacokinetics and tolerability of the novel tubulin targeted agent vinflunine (VFL) (320 mg m^−2^ q 21 days) as second-line chemotherapy in patients with metastatic breast carcinoma (MBC). All patients had disease progression after anthracycline/taxane (A/T) therapy. They could have received a nonanthracycline adjuvant treatment and subsequently received a first-line A/T combination for advanced/metastatic disease; or relapsed >6 months after completion of adjuvant A/T therapy and were subsequently treated with the alternative agent; or relapsed within 6 months from an adjuvant A/T combination. Objective response was documented in 18 of 60 patients enrolled (RR: 30% (95% confidence interval (CI): 18.9–43.2%)). Among the responders, seven patients had relapsed during a period of <3 months from taxane-based regimen yielding a RR of 33.3%. The median duration of response was 4.8 months (95% CI: 4.2–7.2), median progression-free survival was 3.7 months (95% CI: 2.8–4.2) and median overall survival was 14.3 months (95% CI: 9.2–19.6). The most frequent adverse event was neutropenia (grade 3 in 28.3% and grade 4 in 36.7% of patients). No febrile neutropenia was observed. Fatigue (grade 3 in 16.7% of patients) and constipation (grade 3 in 11.7% of patients) were also common; these were non-cumulative and manageable permitting achievement of a good relative dose intensity of 93.5%. Vinflunine is an active agent with acceptable tolerance in the management of MBC patients previously treated with (A/T)-based regimens. These encouraging phase II results warrant further investigation of this novel agent in combination with other active agents in this setting or in earlier stages of disease.

Anthracyclines, taxanes and vinorelbine are among the most active agents in the management of advanced breast cancer. Despite the high level of activity of these agents, metastatic breast cancer (MBC) is essentially an incurable disease; disease progression during or after chemotherapy is unavoidable (Hamilton and Hortobagyi, 2005). No consensus exists for the use of alternative chemotherapy after anthracycline and taxane failure in MBC; only capecitabine is currently registered as a standard treatment in this setting. Therefore, there is a need for new chemotherapy strategies and new agents in this setting.

Vinflunine (VFL) (Javlor®, Pierre Fabre Médicament, Boulogne-Billancourt, France) is a novel microtubule inhibitor agent obtained by semisynthesis using superacidic chemistry to selectively introduce two fluorine atoms at the 20’ position of catharanthine moiety of Vinca alkaloid (Fahy *et al*, 1997). The diverse actions of VFL on microtubules are likely to produce different effects on mitotic spindle functions leading to modifications of cell cycle progression and cell killing (Ngan *et al*, 2000). Vinflunine prevents microtubule assembly during mitosis and induces apoptosis (Kruczynski *et al*, 1998, 2002). Vinflunine exerts effects on microtubule dynamic instability suppressing the rate and extent of microtubule growing events (Ngan *et al*, 2001). The affinity profile of VFL shows features which suggest that it will have greater effects on mitotic rather than axonal tubulin and so will not cause significant neurotoxicity (Lobert *et al*, 1998). Vinflunine showed definite high antitumour activity compared to vinorelbine in xenografts; against the human MX-1 breast xenografts, VFL produced an overall growth inhibition of 61%, whereas vinorelbine did not result in any significant inhibition (Hill *et al*, 1999; Kruczynski *et al*, 1999). Moreover, in the first Phase I study, two out of three patients with heavily pretreated MBC achieved partial response (PRs), therefore, VFL was considered to be a good candidate for further investigation in patients with MBC. Vinflunine treatment every 3 weeks was considered optimal, based on clinical and pharmacokinetic data from the three dose schedules evaluated in Phase I trials. Vinflunine was administered at the beginning of the phase I trials at 350 mg m^−2^ in normal saline as a 10-min infusion; after an early safety analysis this dose was adjusted to 320 mg m^−2^ which is the recommended dose for all subsequent patients included in clinical trials (Bennouna *et al*, 2003).

## MATERIALS AND METHODS

### Objectives

This study was a multicentre, international, nonrandomised Phase II trial, designed to determine the objective response of intravenous VFL administered once every 3 weeks in patients with MBC who had received prior anthracycline/taxane (A/T)-based regimens. Secondary objectives were to assess duration of response, progression-free survival (PFS) and overall survival (OS) and to evaluate the treatment-related toxicity. This trial also included assessment of the pharmacokinetic profile of VFL and planned to document pharmacokinetic/pharmacodynamic relationships in a subset of patients.

The protocol and its amendments were submitted to Independent Ethics Committees and Health Authorities according to local requirements. The study was conducted in accordance with the principles set forth in the Declaration of Helsinki (Somerset West October 1996) and in compliance with Good Clinical and Laboratory Practices. Written informed consent was obtained from each participating patient before enrolment.

### Patient selection

Patients were recruited from 15 active centres between March 2002 and September 2003. Patients were required to have histologically proven breast adenocarcinoma with documented progressive disease. Eligibility also included either (1) nonanthracycline adjuvant treatment with at least 6 months disease-free interval and subsequent treatment with a first-line A/T combination for advanced/metastatic disease; or (2) disease relapse more than 6 months after completion of adjuvant anthracycline-containing chemotherapy and subsequent therapy with a taxane-containing combination as first-line treatment for advanced/metastatic disease; or (3) relapsed more than 6 months after completion of adjuvant taxane-containing chemotherapy and subsequently treated by an anthracycline-containing combination as first-line treatment for advanced/metastatic disease or (4) relapsed within 6 months from an adjuvant A/T combination therapy. The presence of at least one bidimensionally measurable lesion, not previously irradiated, and assessed by CT-scan was required; lesions were documented by WHO criteria. Patients were required to be women, aged ⩾18 years with Karnofsky Performance Status (KPS) ⩾80% and an estimated life expectancy of ⩾12 weeks with adequate haematological function (absolute neutrophil count ⩾2.0 × 10^9^ l^−1^, platelets ⩾100 × 10^9^ l^−1^), hepatic function (bilirubin ⩽1.5 × upper normal limit (UNL), transaminases ⩽2.5 × UNL, unless due to liver involvement), renal function, and a normal electrocardiogram.

### Treatment schedule

Vinflunine was administered at the dose of 320 mg m^−2^ in normal saline as a 10-min infusion every 21 days. The use of haematopoietic growth factors (G-CSF) was allowed for patients with febrile neutropenia or neutropenic infections according to institutional standard and practice.

Vinflunine was delayed by 1 or 2 weeks in case of >grade 2 haematological or nonhaematological toxicity. If febrile neutropenia and/or grade 4 neutropenia (<1.0 × 10^9^ l^−1^) lasting 7 days or more was observed between two subsequent administrations of VFL, the dose was to be reduced to 280 mg m^−2^ from the next cycle on. If, after a dose reduction, this toxicity was seen again, the dose was to be further reduced to 250 mg m^−2^. If at this dose level the same event recurred, the treatment was to be discontinued. No dose re-escalation was allowed after dose reduction. In case of grade 2 mucositis and/or constipation lasting >5 days, or grade ⩾3 mucositis, and/or constipation of any duration, the VFL dose was to be reduced to 280 mg m^−2^ from the next cycle on. If, after dose reduction, one of these toxicities was seen again, the dose was to be reduced to 250 mg m^−2^. If at this dose the event recurred, the treatment was discontinued.

Tumour response was assessed after the initial two cycles of therapy. Patients with complete response (CR) or a PR could continue treatment either until progressive disease (PD), toxicity or patient preference precluded further therapy; those with stable disease (SD) received two additional cycles, were assessed a second time and treatment was continued according to the Investigator's opinion. Patients with PD discontinued the treatment.

### Baseline and treatment evaluation

Baseline assessments included a detailed medical history; computed tomography and physical examination (superficial lymph node or skin nodule). All positive imaging procedures at study entry had to be repeated every 6 weeks. An assessment of symptoms was made at study entry and then throughout treatment. Physical examination and vital signs were assessed on day one of each 3-week cycle. A complete blood cell count was taken at baseline and before each cycle of treatment. Additional samples were planned on day 8 and 15 of each cycle: in case the ANC was <1.0 × 10^9^ l^−1^, counts were repeated every 2 days until recovery to ANC ⩾1.0 × 10^9^ l^−1^. Transaminases, alkaline phosphatases, total bilirubin, LDH, creatinine, electrolytes including Ca^2+^, Na^+^ and K^+^ and total protein were assessed at every cycle. Electrocardiogram was to be performed and recorded before initial administration and repeated at every cycle.

Response was assessed using the WHO criteria (World Health Organization, 1979; Green and Weiss, 1992). In order to be confirmed, CR and PR were to be maintained at 4 weeks. An independent radiologist reviewed all responses and disease stabilisations. According to WHO criteria, the duration of response was calculated for patients with confirmed response (CR or PR) from the date of registration until the date of documented progression, start of new anticancer therapy, date of death, loss to follow-up or last news. The PFS was defined as the time elapsed from registration until progression, death, loss to follow-up or last news; OS was defined as the time elapsed from registration date to death, loss to follow-up or last news. Safety analyses were performed in patients having received at least one dose of study treatment according to the NCI CTC Version 2.0 grading.

### Pharmacokinetic sampling

Pharmacokinetic sampling of VFL was optional and was studied during cycles 1 and 2. Three blood samples were collected between T0 and T16 (initial 16 h). The exact time of sampling was randomly assigned to the investigator centres in order to give wide coverage of the time interval. Vinflunine and 4-*O*-deacetylvinflunine (DVFL) blood concentrations were quantified using a HPLC method with UV detection.

### Statistical analysis

A total of sample size of 55 evaluable patients was calculated using a one-sample multiple testing procedure (Fleming, 1982) and was based on a null hypothesis for the true response rate of 15% and an alternative hypothesis of 30% with a type I error alpha of 5% and type II error beta of <20%. Continuous data were summarised using median, minimum and maximum values. Categorical data were presented in contingency tables with frequencies and percentages. Confidence intervals (CI) were calculated at the 95% level. Time dependent parameters were analysed using the Kaplan–Maier method and 95% CI of the median was reported.

Efficacy analyses were performed on the intent to treat (ITT) and evaluable populations. The other efficacy parameters were duration of response, PFS and OS. A subgroup analysis of response was performed according to the time elapsed between the end of treatment with the taxane-containing regimen and progression. Safety analyses were performed in patients having received at least one dose of study treatment for haematological and nonhaematological adverse events.

All statistical analyses were performed with the 8.2 version of SAS® (SAS Institute Inc., Cary, NC USA) for Windows® .

## RESULTS

Sixty patients with advanced or metastatic carcinoma of the breast were enrolled. Among these patients, five were not eligible but considered for the study evaluation on an ITT basis (one with no target lesions, one without prior chemotherapy for advanced disease, one patient with more than one prior line of chemotherapy, one received cisplatin in the first-line setting but received A/T in the adjuvant setting; one patient assessed without imaging procedures).

Demographic features of the patients are summarised in Tables 1 and 2. All patients enrolled had received prior A/T-based chemotherapy before study entry in neoadjuvant intent for one patient (1.7%), adjuvant intent for two patients (3.3%) and as first-line treatment for advanced disease in 57 patients (95.0%) (Tables 3 and 4).

The median time elapsed between diagnosis of invasive breast cancer and inclusion in the trial was 4.2 years (range: 0.4–17.3). The median progression-free interval after taxane-based regimens was 4.8 months (range: 0–29).

The first stage required five of 30 patients to have an objective response (CR+PR). As nine of the first 30 patients (30%) met this criterion, the study was opened to the second stage and an additional 25 patients were enrolled.

Among the 60 patients included, 18, achieved a tumour objective response of 30% (95% CI: 18.9–43.2%]), and 21 had a stable disease of 35.0% (Table 5).

Of note, 14 of the responders had visceral involvement (45 patients had visceral involvement at baseline). Five patients could not be evaluated for response but were kept in the denominator for the purpose of the calculation of the response rate: three patients received a single cycle of treatment (one patient due to drug-related toxicity, one case of patient's refusal to continue and one case due to nondrug-related adverse event), one unevaluable due to the fact that CT scan slides were not comparable over the different tumour assessments, and one patient with liver haemangioma as selected target lesion (after panel review).

It is noteworthy that among the 18 responders seven had experienced previous tumour relapse <3 months, achieving a RR of 33.3% (95% CI: 14.6–57.0) in this group.

**Figure 1:**
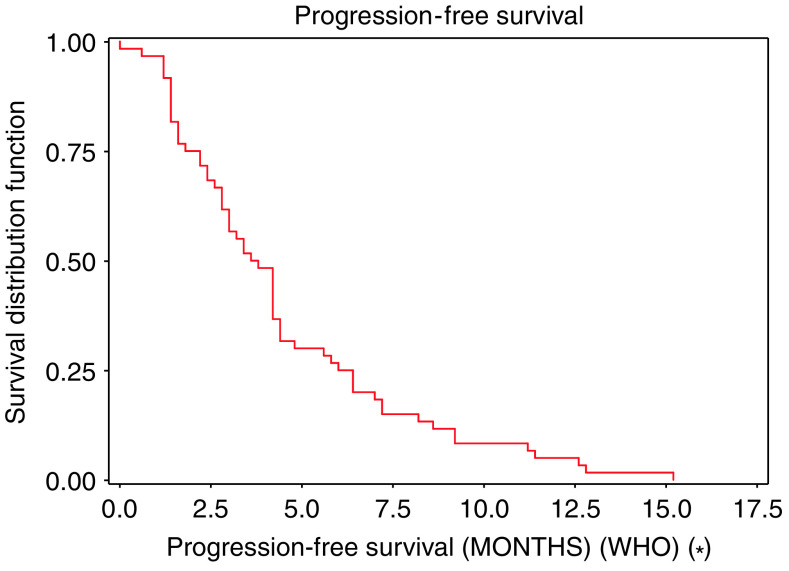
Progression-free survival.

**Figure 2:**
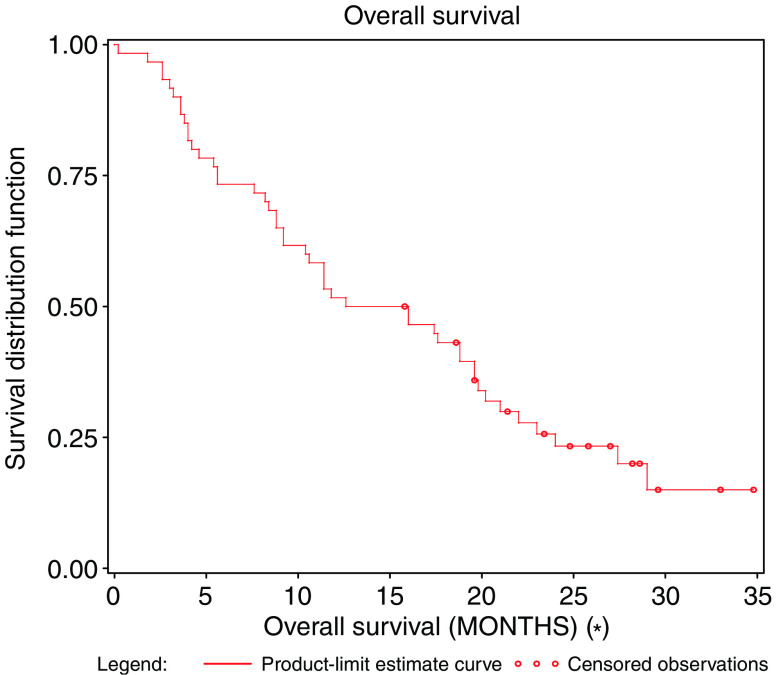
Overall survival.

The median duration of response was 4.8 months (95% CI: 4.2–7.2). The median duration of stable disease was estimated at 4.1 months (95% CI: 3.3–5.7). Median PFS was 3.7 months (95% CI: 2.8–4.2) (Figure 1), and the median OS was estimated at 14.3 months (95% CI: 9.2–9.6) (Figure 2).

Karnofsky Performance Status (KPS) was recorded for each patient at baseline and before each treatment cycle during the study (every 3 weeks). During treatment the KPS stayed the same or improved in 43 out of 60 patients; only two patients experienced a decrease of 20% in KPS score.

### Pharmacokinetic results

The number of patients evaluable for pharmacokinetics was 17 and 15 in cycles 1 and 2, respectively. All patients were first treated at the dose of 320 mg m^−2^, seven of them had a dose reduction to 280 mg m^−2^ in cycle 2; results are shown in Table 6.

The mean total clearance and the range of the individual values were comparable between cycles 1 and 2. The intraindividual variability of Cl_tot_ was low between cycles 1 and 2: variability values were all <19% and 11 out 15 values were <10%. No statistically significant difference in Cl_tot_ (*P*=0.871) and *T*_1/2 z_ (*P*=0.928), was observed between cycles 1 and 2 using a paired *t*-test. The four patients who experienced grade 3 and 4 SAEs, did not show any VFL blood overexposure.

### Safety evaluation

All 60 patients were evaluable for safety. The total number of cycles administered was 292; (median 5 cycles; (range 1–12)). The median relative dose intensity was 93.5%. Twenty-two out of the 60 patients (36.7, 9.9% of cycles) had dose reductions.

Twenty-four patients (40%) experienced grade 3/4 leucopenia during the study (15.2% of cycles). Grade 3/4 neutropenia occurred in 65% of patients (36.2% of cycles) (Table 7). The median nadir value for leucocytes and neutrophils was 2.3 × 10^9^ l^−1^ and 0.7 × 10^9^ l^−1^, respectively. The median cycle at which both leucocyte and neutrophil nadirs occurred was the second cycle of treatment (on days 8 and 11, respectively). The median duration of leucocyte and neutrophil nadir was 7 days. No febrile neutropenia was observed. Grade 3–4 neutropenia complicated with infection was observed in 8.3% of patients (1.7% of cycles). Only 3.1% of patients received growth factor support.

Anaemia was rarely severe; only 5.0% of patients developed grade 3 toxicity (1.0% of cycles). The incidence of thrombocytopenia was low, only one out of 60 patients developed grade 3 (0.3% of cycles).

The incidence (Table 5) of severe nausea and vomiting was very low: only 8.3% of patients experienced grade 3 nausea and only 6.7% of patients experienced grade 3 vomiting. The overall incidence of constipation was 63.3%; 16 patients (26.7%) requiring stool softeners or dietary modifications (grade 1), four (6.7%) requiring laxatives (grade 2) and seven patients (11.7%) experienced grade 3 constipation (2.7% of cycles) requiring enema. Ileus was reported in two patients, this event occurred after the first administration and recovered completely allowing continuation of treatment at 280 mg m^−2^.

Grade 3 or 4 myalgia was observed in only 3.4% of patients (0.6 % of cycles). Grade 3 fatigue was experienced by 16.7% of patients (4.1% of cycles). No grade 3–4 peripheral neuropathy was experienced. Among the 36 patients receiving the drug via peripheral veins, 13 developed mild injection site reactions of grade 1 or 2; no grade 3 reactions were observed. Grade 1–2 alopecia was seen in 35% of patients. No clinically significant alterations in renal or hepatic function were observed. No treatment-related deaths were reported.

## DISCUSSION

The data of our study support the hypothesis that VFL is an active and well-tolerated agent for the treatment of patients with MBC. Specifically, these data were derived in the setting of prior therapy with anthracycline and taxane, a situation that is increasingly common in breast cancer management. The value of identifying novel agents for the management of MBC patients previously treated with anthracyclines and taxanes is underscored by the fact that in the eligible patient population only capecitabine is currently registered as a standard treatment in this setting.

The first trial to assess capecitabine was conducted in 162 patients with paclitaxel-refractory MBC; the ORR in this population was 20%, median time to progression (TTP) 3 months, and OS 12.6 months (Blum *et al*, 1999). An ORR of 15% was obtained in another clinical study (136 patients), with a median TTP and OS of 3.5 months and 10.1 months was observed respectively; the median interval between the last taxane-containing therapy and start of capecitabine treatment in this study was 4.4 months and 17% of patients discontinued the study drug due to drug-related adverse events, mainly hand-foot syndrome (Reichardt *et al*, 2003). Most recently, in a study of 126 patients with MBC treated with capecitabine after two or three prior chemotherapies including an anthracycline and a taxane, the ORR was 28.0% and the TTP was 4.9 months (95% CI: 4.0–6.4 months) with a median OS of 15.2 months (95% CI: 13.5–19.6 months) (Fumoleau *et al*, 2004). One single-agent study with gemcitabine at 1200 mg m^−2^ days 1, 8 and 15 q 4 weeks, failed to demonstrate objective response in 23 patients (Smorenburg *et al*, 2001). Clearly other active agents are needed.

A phase II trial of vinorelbine in 40 patients with paclitaxel-refractory MBC demonstrated a median time to progression of 3 months and a median survival of 8 months with an objective response rate of 25% (Livingston *et al*, 1997). Moderate activity has been observed with irinotecan and pemetrexed yielding ORRs of 14–26% and 10–26%, respectively, in patients with MBC after anthracycline and/ or taxane exposure (Calvert, 2003; Heinemann, 2003; Perez *et al*, 2004). In a phase II trial with ixabepilone, 37 women with metastatic and locally advanced breast cancer who had received paclitaxel and/or docetaxel as prior neoadjuvant, adjuvant or metastatic therapy, the ORR was 22% and median duration of response 3.8 months; the median PFS was established at 2.8 months (Low *et al*, 2005).

The present study confirms that VFL is an active and tolerable treatment for the management of MBC previously treated with anthracycline and taxane-based regimens. The overall response rate of 30% and the response rate of 33% in patients who had developed tumour relapse less than 3 months after a taxane containing regimen, place VFL among the most active drugs in this setting. This is supported by the median OS estimated at 14.3 months. Although VFL was associated with some severe toxicity in the form of myelosuppression and constipation these adverse events were predictable, reversible, noncumulative and manageable.

In conclusion, the present study demonstrates that VFL has clinically relevant activity with acceptable toxicity in the treatment of patients with advanced breast cancer who have failed prior A/T containing regimens. These results warrant further investigation in phase III trials of MBC either as monotherapy or in combination with other agents in first-line treatment.

## Figures and Tables

**Table 1:** Demographic data

**Demographic data**	**ITT patients**
*Age (years) (N*=*60*)	
Median	55.2
Range	(33.0–75.8)
	
*Karnofsky performance status* (%)	*N*=60 (%)
100	18 (30.0)
90	19 (31.7)
80	20 (33.3)
70	3 (5.0)
	
*No. of organs involved*	
1	16 (26.7)
2	23 (38.3)
⩾3	21 (35.0)
	
*Organs involved*	
Liver	40 (66.7)
Lung	19 (31.7)
Lymph nodes	24 (40.0)
Pleural effusion	13 (21.7)
Soft tissue	8 (13.3)
Skin	8 (13.3)
Others	6 (10.0)
Breast	1 (1.7)
Bone	21 (35.0)

Abbreviation: ITT=intent to treat.

**Table 2:** Hormonal receptors at diagnosis

	**ER**			
	**Positive**	**Negative**	**Unknown or missing**	**Total**
*PR*				
Positive	22	2	0	24
Negative	7	13	0	20
Unknown or missing	9	0	7	16
Total	38	15	7	60

Abbreviation: PR=partial response.

**Table 3:** Prior chemotherapy

	**(*N*=60)**	**%**
*Intent of prior chemotherapy*		
Neoadjuvant	1^a^	1.7
Adjuvant	2^a^	3.3
Neoadjuvant+metastatic	5	8.3
Adjuvant+metastatic	35	58.3
Neoadjuvant+adjuvant+metastatic	2	3.3
Metastatic	15	25

a Two patients were enrolled into the study after adjuvant or neoadjuvant failure; relapsing within 6 months of last administration of neoadjuvant anthracycline and taxane combination.

**Table 4:** Previous chemotherapy for advanced disease

	***N* (%)**	**%**
*Class of chemotherapy*		
Taxane-anthracyclines	31^a^^,^^b^	51.6
Taxane single agent	21	35.0
Taxane+other	3	5.0
Anthracycline based	4	6.7
Cisplatin	1	1.7

a Two patients were enrolled into the study after adjuvant or neoadjuvant failure; relapsing within 6 months of last administration of neoadjuvant anthracycline and taxane combination.

b One patient having progressed >6 months after adjuvant treatment (considered a major protocol deviation).

**Table 5:** WHO overall response rates

**Overall response**	**ITT *N*=60 (%)**
PR	18 (30.0)
95% CI	(18.9–43.2)
Stable disease	21 (35.0)
Nonevaluable	5 (8.3)
Disease progression	16 (26.7)

Abbreviations: CI=confidence interval; PR=partial response; ITT=intent to treat; WHO=World Health Organization.

**Table 6:** Main mean (s.d.) blood pharmacokinetic parameters of vinflunine

		**Cycle 2 (*n*=15 patients)**
	**Cycle 1 (***n***=17 patients)**	**320 mg m^−2^: 8 patients**
**Parameters**	**320 mg m^−2^**	**280 mg m^−2^: 7 patients**
Cl_tot_ (L.h^−1^)	41.7 (12.1)	42.5 (14.4)
T_1/2__z_ (h)	46.6 (7.58)	46.4 (7.73)
Vd (L)	2749 (269)	2770 (306)

**Table 7:** Adverse events according to NCI CTC (version 2.0)

	***N*=60 patients**			***N***=**288^a^ cycles**		
**Adverse events (%)**	**Overall incidence**	**Grade 3**	**Grade 4**	**Overall incidence**	**Grade 3**	**Grade 4**
Anaemia	50 (83.3)	3 (5.0)	0	180 (62.5)	3 (1.0)	0
Leucopenia	55 (91.7)	21 (35.0)	3 (5.0)	207 (71.9)	41 (14.2)	3 (1.0)
Neutropenia	55 (91.7)	17 (28.3)	22 (36.7)	211 (73.3)	69 (24.0)	35 (12.2)
Thrombocytopenia	30 (50.0)	1 (1.7)	0	56 (19.4)	1 (0.3)	0
						
Febrile neutropenia	0	0	0	0	0	0
						
*Infection*						
Infection+G 3/4 neutropenia	5 (8.3)	3 (5.0)	1 (1.7)	5 (1.7)	3 (1.0)	1 (0.3)
Infection without neutropenia	3 (5.0)	0	0	4(1.4)	0	0
						
*Gastrointestinal*						
Nausea	35 (58.3)	5 (8.3)	0	79 (27.1)	5 (1.7)	0
Vomiting	28 (46.7)	4 (6.7)	0	54 (18.5)	5 (1.7)	0
Constipation	38 (63.3)	7 (11.7)	0	89 (30.5)	8 (2.7)	0
Diarrhoea	8 (13.3)	1 (1.7)	0	10 (3.4)	1 (0.3)	0
Stomatitis	33 (55.0)	1 (1.7)	0	54 (18.5)	1 (0.3)	0
						
*Dermatological*						
Alopecia	21 (35.0)	NA	NA	NA	NA	NA
Injection site reaction	13 (36.1)	0	NA	24 (21.8)	0	NA
						
*Neurology*						
Ileus	2 (3.3)	2 (3.3)	0	2 (0.7)	2 (0.7)	0
Neuropathy sensory	8 (13.3)	0	0	22 (7.5)	0	0
						
*Pain*						
Abdominal pain	27 (45.0)	7 (11.7)	0	45 (15.4)	8 (2.7)	0
Arthralgia	14 (23.3)	1 (1.7)	0	30 (10.3)	1 (0.3)	0
Myalgia	14 (23.3)	1 (1.7)	1 (1.7)	21 (7.2)	1 (0.3)	1 (0.3)
Jaw pain	8 (13.3)	2 (3.3)	0	30 (10.3)	9 (3.1)	0
						
*Flu-like symptoms*						
Fatigue	49 (81.7)	10 (16.7)	0	147 (50.3)	12 (4.1)	0
Fever without neutropenia	6 (10.0)	0	0	6 (2.1)	0	0

a Four cycles were not assessed for haematological parameters due to missing data.
